# Phenoxodiol protects against Cisplatin induced neurite toxicity in a PC-12 cell model

**DOI:** 10.1186/1471-2202-8-61

**Published:** 2007-08-01

**Authors:** Reuben Klein, David Brown, Ann M Turnley

**Affiliations:** 1 Centre for Neuroscience, The University of Melbourne, Parkville, Victoria 3010 Australia; 2 Novogen Ltd, 140 Wicks Road North Ryde NSW, Australia

## Abstract

**Background:**

Many commonly used chemotherapeutic agents, such as Cisplatin, are restricted in their potential anti-neoplastic effectiveness by their side effects, with one of the most problematic being induction of peripheral neuropathy. Although a number of different neurotrophic, neuroprotective or anti-oxidant treatments have been tried in order to prevent or treat the neuropathies, to date they have met with limited success. Phenoxodiol is a new chemotherapeutic agent that has anti-proliferative and apoptotic effects on a range of cancer cells. PC12 cells are a commonly used neuronal cell model for examination of neurite outgrowth. In this study we examined whether phenoxodiol could protect against Cisplatin induced neurite inhibition in PC12 cells as an indication of the potential to protect against neuropathy.

**Results:**

Using the PC12 neuronal cell line, concentrations of Cisplatin were chosen that induced moderate or strong neurite toxicity within 24 hrs but were not cytotoxic. The effect of Phenoxodiol on Cisplatin induced neurite toxicity was assessed by measurement of neurite outgrowth. Addition of phenoxodiol at 100 nM or 1 μM showed no cytotoxicity and blocked the Cisplatin induced neurite toxicity, while phenoxodiol at 10 μM was cytotoxic and enhanced neurite toxicity of Cisplatin. When Cisplatin was added for 24 hrs, then washed out and the cells allowed to recover for 48 hrs, neurite outgrowth was not restored and addition of phenoxodiol did not further promote recovery or restore the Cisplatin treated cells.

**Conclusion:**

In addition to its potential as a chemotherapeutic agent Phenoxodiol may thus also have the potential to be used in conjunction with Cisplatin chemotherapy to prevent induction of neuropathy.

## Background

Many common chemotherapeutic drugs are limited in their effectiveness due to side effects such as peripheral neuropathy. This is particularly problematic for use of otherwise highly effective anti-neoplastic agents, such as platinum analogues or taxane family members, as the effects are often dose or treatment regime limiting. The neurotoxic effects can be severe and significantly affect quality of life, even long after the treatment has ceased [1]. Even though there is often some regeneration, this is slow and in many instances the reversal of the neuropathy is incomplete and can affect quality of life and normal function for many years.

The platinum analogue Cisplatin (*Cis*-diamine-dichloro-platinum) has been used chemotherapeutically for nearly 40 years and is one of the most widely used cytotoxic drugs [1]. Cisplatin is directly taken up by sensory nerves. Although Cisplatin produces its anti-neoplastic effects by binding directly to DNA, resulting in cross-linking and production of apoptosis in rapidly dividing cells [2], as neurons are post-mitotic, the mechanism by which it induces neuropathy is not clear. While it does appear to induce apoptosis in sensory neurons [3], early stages involve axonal loss but not necessarily cell loss and has been proposed to involve a disturbance of cytoplasmic/axonal transport [1].

Many compounds have been tested to try to block or reverse these chemotherapy induced neuropathies, with variable success. These include neurotrophic or neuroprotective factors, such as nerve growth factor (NGF) [4,5], insulin-like growth factor-1 (IGF1) [6], erythropoietin [7,8] and leukaemia inhibitory factor (LIF) [9], all of which showed some limited improvement in a variety of models. Their clinical use is however limited due to difficulties in drug administration, stability, deleterious side effects or ineffectiveness in human clinical trials [10]. Antioxidants such as glutathione [11] and Vitamin E [12] and neuroprotective compounds such as acetyl-L-carnitine [13,14] have also shown some effectiveness in protecting against chemotherapy induced neuropathy in preliminary studies. However, to date, there is no compound that will reliably prevent or reverse such neuropathies.

Phenoxodiol (PXD; 2H-1-benzopyran-7-0,1,3-[4-hydroxyphenyl]) is an isoflavone analogue derived from genistein, which shows greater bio-availability and increased potency than its parent compound. It is showing promise as an experimental chemotherapeutic drug [15,16] and is currently undergoing phase II clinical trials for the treatment of a variety of hormone-resistant cancers. It induces apoptosis in a variety of cancer cell lines [15-17] by modulation of a number of apoptotic pathways including activation of caspase 2 and Bid signalling and inhibition of phosphorylation and degradation of the anti-apoptotic protein XIAP [17,18]. It also shows anti-angiogenic properties [16] and anti-proliferative properties by inducing G1 arrest by loss of cyclin dependent kinase 2 activity and induction of p21 Waf1/Cip1 [19]. While it shows cytotoxicity by itself, it also sensitises cancer cells to chemotherapeutic agents such as Paclitaxel [18], allowing chemotherapy resistant tumours to become responsive. PXD thus has a range of biological effects and more are likely when examined in different cells and in different contexts.

In the current study the potential for PXD to protect against Cisplatin induced neuropathy was assessed. Using an in vitro model, PXD at sub-toxic concentrations was shown to be effective at blocking Cisplatin induced neurite toxicity in the neuronal PC12 cell line. This suggests that in addition to its antineoplastic properties, PXD has the potential to protect against Cisplatin induced neuropathy.

## Methods

### PC12 cell culture

PC-12 cells were maintained in Dulbecco's Modified Eagle Media (DMEM; Gibco) with 10% calf serum (Turbo calf serum, Invitrogen), 5% horse serum (JRH Biosciences, Victoria, Australia) and 1% penicillin/streptomycin (Invitrogen) at 37°C, in a 5% CO_2 _atmosphere. Differentiation into neurons was achieved by seeding cells at a density of 15,000 cells/well in 24-well plates (Falcon Becton Dickinson), on 13 mm glass cover slips (Menzel Glaser, Germany) coated with laminin (Invitrogen) and poly-DL-ornithine (Sigma) in DMEM plus 1% horse serum and 50 ng/mL of nerve growth factor (NGF) (Sigma). The cells were incubated for 72 hours under differentiation conditions, as previously described [20] before use in the neurite outgrowth assays below.

### Cisplatin and PXD Treatments

Cisplatin (Sigma) and PXD were provided by Novogen. They were dissolved in DMSO and stocks maintained at -20°C. Working concentrations were diluted in PC12 cell differentiation medium as above. Control conditions contained DMSO diluted as for the drug conditions.

Combination treatments consisted of a 24 hour incubation in differentiation media with various combinations of strong or moderate doses of Cisplatin (20 μg/ml/66.65 μM and 1 μg/ml/3.33 μM) and three doses of PXD (100 nM, 1 μM, 10 μM) as described in the results section. Recovery treatments consisted of treating differentiated cells with either dose of Cisplatin for 24 hours. After this period, the cells were allowed to recover for 24 hours in fresh differentiation media. Finally, three concentrations of PXD were added (10 nM, 100 nM, 1 μM) for a further 24 hours to assess whether PXD could rescue the neurites from the Cisplatin induced damage.

### Immunocytochemistry

Following the treatment period, cells were washed with PBS, fixed with 4% paraformaldehyde and permeabilised with ice-cold methanol. Neurons were immunostained for the neuronal marker βIII-tubulin (Promega; Madison, Wisconson, USA) and a Cy3-conjugated anti-mouse antibody (Invitrogen) was used to visualise the staining.

### Neurite counting

Outgrowing projections were considered neurites if they were greater than a cell body width (10 μm), essentially as previously described [21]. Images of all neurons in thirty randomly selected fields were digitally captured using an Olympus BX60 fluorescence microscope with a UPlanFl 40x/0.75 objective lens and a Zeiss Axiocam HRc digital camera and Zeiss Axiovision 3.1 software. Neurons in all fields were counted per condition and the number of cells with neurites expressed as a percentage of total cells present and error expressed as the standard error of the mean (sem). Each experiment was conducted in triplicate and the results pooled. Statistical significance of data was analysed using ANOVA followed by the Bonferroni *post-hoc *test.

Further analysis was conducted on the combination treatments, where the longest neurite per neuron present in each frame was measured using Image J open source software (NIH, USA).

## Results

### Determination of drug doses

**Figure 1:**
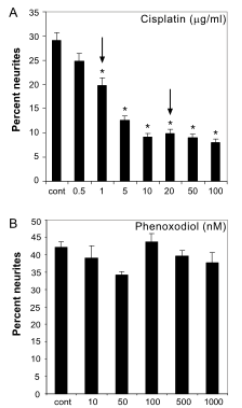
**Cisplatin and PXD neurite toxicity dose response**. The percentage of differentiated PC12 cells with neurites was counted after incubation for 24 hrs in increasing concentrations of A) Cisplatin (* *p *< 0.001 compared to control) and B) PXD. Cisplatin showed a dose response in inhibition of neurite outgrowth and moderate and strong concentrations of each indicated by arrows, were chosen for subsequent analyses.

Optimal treatment concentrations Cisplatin that caused neurite damage were determined by treating differentiated PC12 cells for 24 hours with serial dilutions of the drug. The concentration which caused the greatest reduction in the percentage of cells with neurites, without killing the cells, was chosen as a strong dose. A moderate dose was also selected, where the drug reduced neurite outgrowth by approximately 50% of the strong dose. The strong dose of Cisplatin was selected as 20 μg/mL (66.65 μM), which reduced the percentage of cells with neurites by 65.8%, and the moderate dose of 1 μg/mL (3.33 μM) caused a 31.9% reduction in percentage of cells with neurites (Fig. 1a). The chosen concentrations did not produce cytotoxicity (data not shown).

A serial dilution was also conducted for PXD to determine the concentration which would not affect normal growth of the differentiated cells. In addition to the maximal concentration tested that did not affect survival of PC12 cells (1 μM; 3.2 μg/ml), two other concentrations were selected for treatments, one log above (10 μM; 32 μg/ml) and one log below (100 nM; 320 ng/ml). Doses up to 1 μM had no effect on cell death or neurite outgrowth (Fig. 1b), although a 10 μM concentration showed considerable cytotoxicity (data not shown).

### Effect of PXD in blocking Cisplatin neurite toxicity

**Figure 2:**
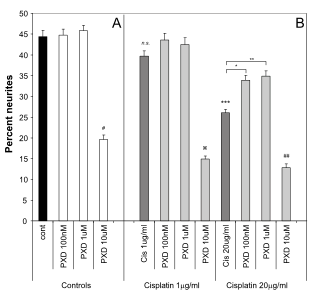
**Protection from Cisplatin induced neurite toxicity by PXD**. Differentiated PC12 cells were incubated with Cisplatin alone or in combination with PXD for 24 hrs and the percentage of cells with neurites was determined. A) PXD alone had no effect on neurite outgrowth up to a concentration of 1 μM, however 10 μM PXD showed neurite toxicity (^#^*p *< 0.001). B) Cisplatin (1 μg/ml) showed a non-significant (n.s.) trend for moderate toxicity and at 20 μg/ml showed strong neurite toxicity (***p < 0.001). PXD blocked the Cisplatin induced neurite toxicity at 100 nM (*p *< 0.01) and 1 μM (*p *< 0.001), while the combination of 10 μM PXD and Cisplatin at 1 μg/ml or 20 μg/ml showed enhanced neurite toxicity compared to 10 μM PXD alone (*p *< 0.01 and ^##^*p *< 0.001 respectively, combined results of *n *= 3 experiments).

To determine whether PXD could block Cisplatin induced neurite toxicity, three different concentrations of PXD were added to cells in combination with Cisplatin at the concentrations determined above to produce strong or moderate neurite toxicity. PXD had no effect on percent neurites at 100 nM or 1 μM but showed significant neurite toxicity at 10 μM (# *P *< 0.001 compared to no treatment control) (Fig. 2a). This neurite toxicity was exacerbated in combination with Cisplatin, with increased toxicity compared to PXD at 10 μM alone (z *P *< 0.01 in combination with Cisplatin 1 μg/ml; *## P *< 0.001 in combination with Cisplatin 20 μg/ml) (Fig. 2b).

**Figure 4:**
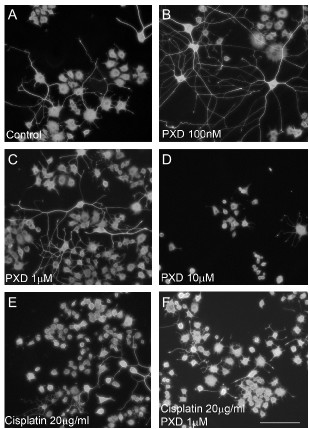
**Effect of PXD Cisplatin on βIII-tubulin stained PC12 cells**. Differentiated PC12 cells were incubated with Cisplatin alone or in combination with PXD for 24 hrs then fixed and immunostained for the neuronal marker βIII-tubulin. A) Control cultures showed a large percentage of cells with long neurites, which were also present in B) 100 nM PXD and C) 1 μM PXD. D) Significant cell and neurite toxicity was observed in cells incubated with 10 μM PXD. E) Cisplatin at 20 μg/ml decreased the percentage of cells bearing neurites compared to control, which was blocked by (F) 1 μM PXD. Scale bar in F represents 50 μm and applies to all panels in the figure.

Although in the initial dose response experiments Cisplatin at 1 μg/ml produced a modest neurite toxicity, this was not robustly observed when subsequent experiments were performed (the effect was not significant when all data was analysed using ANOVA with a Bonferroni *post-hoc *test, although a direct comparison of control versus Cisplatin 1 μg/ml using the *t*-test was significant; *P *< 0.02) (Fig. 2b). Consequently, although there appeared to be a slight protection by PXD, this was not significant by ANOVA, although comparison of Cisplatin 1 μg/ml with Cisplatin 1 μg/ml+PXD 1 μM was significant by *t*-test (*P *< 0.02). Robust neurite toxicity was observed with Cisplatin at 20 μg/ml, with a 42% decrease in percent neurites compared to control (*** *P *< 0.001). PXD blocked this neurite toxicity by approximately 50% at 100 nM (* *P *< 0.01) and 1 μM (** *P *< 0.001) (Fig. 2b; Fig. 4).

### Effect on neurite length

**Figure 3:**
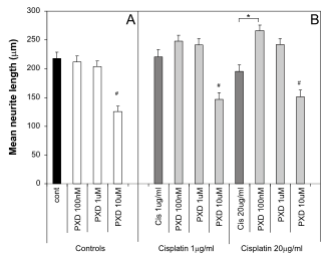
**Effect of Cisplatin and PXD on neurite length**. Differentiated PC12 cells were incubated with Cisplatin alone or in combination with PXD for 24 hrs and the neurite length was determined by measuring the longest neurite on cells with neurites longer than 10 μm. A) PXD alone had no effect on neurite length up to a concentration of 1 μM, however 10 μM PXD decreased neurite length (^#^*p *< 0.001). B) Cisplatin at 1 μg/ml and at 20 μg/ml did not affect neurite length. PXD at 100 nM and 1 μM had little effect on neurite length in the presence of Cisplatin, although there was a slight increase in neurite length with PXD at 100 nM and 20 μg/ml Cisplatin (* *p *< 0.001). The combination of 10 μM PXD and Cisplatin at 1 μg/ml or 20 μg/ml was not different to the effect of 10 μM PXD alone. Data shows combined results of *n *= 3 experiments.

To determine whether there were more subtle effects on neurite toxicity than could be measured by counting the percent of cells with neurite as above, the effect of Cisplatin and PXD on neurite length was examined. The 10 μM concentration of PXD resulted in significant reductions in average neurite length compared to no treatment control, both alone (Fig. 3a) and in combination with Cisplatin (Fig. 3b) (# p < 0.001). While Cisplatin decreased the percentage of cells that had neurites (Figs. 1, 2), it had no significant effect on the average neurite length of the remaining neurites (Fig. 3b). Interestingly, while PDX (100 nM and 1 μM) or Cisplatin alone had no effect on neurite length, the combination of these drugs increased neurite length compared to Cisplatin alone (* p < 0.001), reflecting a blocking of Cisplatin neurite toxicity. Overall, the effect of PXD on blocking Cisplatin effects on neurite outgrowth reflected the observations for the effects on percentage of cells with neurites.

### Effect of PXD in enhancing recovery from Cisplatin neurite toxicity

**Figure 5:**
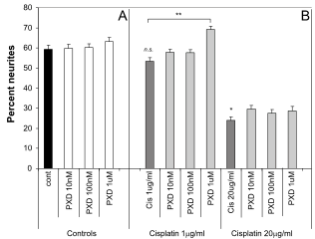
**Lack of rescue of Cisplatin induced neurite toxicity by PXD**. Differentiated PC12 cells were incubated with Cisplatin alone or in combination with PXD for 24 hrs. The cells were washed and left for 24 hrs, then PXD at the concentrations indicated was added for a further 24 hrs before the percentage of cells with neurites was determined. The concentrations of PXD used in these experiments was 1 log lower than in the previous experiments. A) PXD alone had no effect on percent of cells with neurites. B) Cisplatin at 1 μg/ml showed no effect but at 20 μg/ml the cells failed to recover from the neurite toxicity (*p < 0.001 compared to control). PXD did not rescue the cells from the Cisplatin (20 μg/ml) induced neurite toxicity, although it showed 15% enhancement at 1 μM with 1 μg/ml Cisplatin (** *p *< 0.001). Data shows combined results of *n *= 3 experiments.

Co-administration of PXD with Cisplatin blocked the Cisplatin-induced neurite toxicity. To determine whether it could have an effect on recovery from Cisplatin induced neurite toxicity by exacerbation, reversal, retardation or facilitation of repair, recovery experiments were performed. When conducting the recovery experiments, the concentrations of PXD tested were decreased by 1 log to 10 nM, 100 nM and 1 μM so that the highest concentration was below the toxic level. Differentiated PC12 cells were incubated with Cisplatin for 24 hrs as above, which was then washed off and the cells allowed to recover for 24 hrs before PXD was added for a further 24 hrs. Addition of PXD at 10 nM, 100 nM or 1 μM had no effect on neurite outgrowth by themselves (Fig. 5a).

After 48 hrs of recovery, Cisplatin showed no neurite toxicity at 1 μg/ml but greater than 50% toxicity at 20 μg/ml (*P < 0.001) compared to the no treatment control, indicating that the cells could not recover from this higher dose by 48 hrs after drug addition. PXD had no significant effect on recovery of neurite outgrowth following 20 μg/ml Cisplatin treatment, although there was a slight enhancement of neurite outgrowth by PXD (1 μM), following Cisplatin at 1 μg/ml (**P < 0.001) (Fig. 5b). Overall, PXD was protective for Cisplatin induced neurite toxicity at concentrations of 100 nM to 1 μM when co-administered but not when administered after toxicity had already occurred.

## Discussion

PXD at low doses was able to significantly block neurite toxicity induced by Cisplatin in the PC12 neuronal cell model. It did not however, affect recovery from Cisplatin induced neurite toxicity, indicating that PXD needs to be administered prior to or concomitant with Cisplatin to be effective.

The sensitivity of neurite toxicity to the protective effect of PXD was approximately 10 fold higher than the cytotoxic and anti-proliferative effects observed in a variety of cancer cell lines. Significant protective effects of PXD on neurite toxicity were observed at 1 μM, which is within the cytotoxic and anti-proliferative range of PXD in a range of cells including epithelial ovarian carcinoma, human umbilical vein endothelial cells and trophoblast cells [16-18,22]. However, significant neurite protective effects were also observed at a 10 fold lower concentration of PXD, which does not show robust effects on cancer cell viability [17] or endothelial cell proliferation [16]. The 1 μM concentration of PXD was not toxic to the PC12 cells, unlike the ovarian cancer cell lines described [17,18], although this concentration is also not toxic to normal ovarian surface epithelial cells [17].

The ability of PXD to protect against Cisplatin induced neurite toxicity is likely reflected in the mechanism by which it produces neurite toxicity. Cisplatin is a DNA binding drug and the amount of DNA cross-linking correlates with cytotoxicity in cancer cells [23,24], which are particularly susceptible due to high mitotic rate as well as a decreased ability to repair Cisplatin-induced DNA damage [25,27]. The DNA binding of Cisplatin also results in damage to a large number of genes [28], which would have consequences for transcription and consequent general metabolism of the cell. How Cisplatin specifically affects neurite outgrowth is as yet unknown but may be a result of general effects on signal transduction and signaling pathways altered because of DNA damage.

Post-mitotic sensory neurons such as Dorsal root ganglia (DRG) neurons are also particularly susceptible to Cisplatin binding [29] and DNA damage, although this can be rescued by administration of 100 ng/ml NGF [30,31]. Similar to DRG neurons, NGF also blocks Cisplatin induced cytotoxicity of PC12 cells [31]. Hence, in our study in which the PC12 cells were maintained in a differentiated state in the presence of NGF, we were able to isolate the effects of Cisplatin on neurite toxicity from that of cytotoxicity. While the mechanism by which PXD blocked neurite toxicity is at this stage unknown, PXD has previously been shown to upregulate p21 Waf1/Cip1 in a range of cancer cell lines [19]. Upregulation of p21 Waf1/Cip1 is however not only associated with cell cycle regulation, it is also a regulator of neurite outgrowth. In the PC12 cell model, as used in this study, upregulation of p21 Waf1/Cip1 promotes neurite outgrowth [32-34]. It also promotes neurite outgrowth in other neuronal cell lines and primary neurons [35], promotes axonal regeneration after spinal cord injury in rats [36] and regulates radial axon growth and motor function recovery following peripheral nerve injury [37]. Therefore, PXD can actively upregulate a signal transduction pathway that is not only closely associated with mitotic arrest but also with neurite outgrowth.

## Conclusion

Overall, given the dose and treatment limiting neuropathic side effects of Cisplatin, one of the most widely used chemotherapeutic drugs and the lack of any currently available prophylactic treatment or cure, PXD is a promising candidate that warrants further testing. Successful prophylactic treatment of Cisplatin induced neuropathy with PXD could allow more intensive and hence more effective Cisplatin therapy.

## Competing interests

This research was supported by Novogen Ltd as a research contract. DB is an employee of Novogen.

## Authors' contributions

DB conceived of the study and assisted in the design of the experiments. RK performed all the experiments, analysed the data and assisted in preparation of the manuscript. AT designed the experiments, assisted in data analysis and wrote the manuscript. All authors read and approved the final manuscript.
